# TMS disruption of the lateral prefrontal cortex increases neural activity in the default mode network when naming facial expressions

**DOI:** 10.1093/scan/nsad072

**Published:** 2023-11-23

**Authors:** David Pitcher, Magdalena W Sliwinska, Daniel Kaiser

**Affiliations:** Department of Psychology, University of York, Heslington, York YO105DD, UK; School of Psychology, Liverpool John Moores University, Liverpool L3 3AF, UK; Mathematical Institute, Department of Mathematics and Computer Science, Physics, Geography, Justus-Liebig-Universität Gießen, Gießen 35392, Germany; Center for Mind, Brain and Behaviour, Philipps-Universität Marburg, and Justus-Liebig-Universität Gießen, Marburg 35032, Germany

**Keywords:** Superior temporal sulcus, fusiform face area, occipital face area, Amygdala

## Abstract

Recognizing facial expressions is dependent on multiple brain networks specialized for different cognitive functions. In the current study, participants (*N *= 20) were scanned using functional magnetic resonance imaging (fMRI), while they performed a covert facial expression naming task. Immediately prior to scanning thetaburst transcranial magnetic stimulation (TMS) was delivered over the right lateral prefrontal cortex (PFC), or the vertex control site. A group whole-brain analysis revealed that TMS induced opposite effects in the neural responses across different brain networks. Stimulation of the right PFC (compared to stimulation of the vertex) decreased neural activity in the left lateral PFC but increased neural activity in three nodes of the default mode network (DMN): the right superior frontal gyrus, right angular gyrus and the bilateral middle cingulate gyrus. A region of interest analysis showed that TMS delivered over the right PFC reduced neural activity across all functionally localised face areas (including in the PFC) compared to TMS delivered over the vertex. These results suggest that visually recognizing facial expressions is dependent on the dynamic interaction of the face-processing network and the DMN. Our study also demonstrates the utility of combined TMS/fMRI studies for revealing the dynamic interactions between different functional brain networks.

## Introduction

Humans need to recognize and interpret the facial expressions of other people during social interactions. The neural computations that support these cognitive processes have been extensively investigated using functional magnetic resonance imaging (fMRI). These studies have been the basis of theories positing that emotions are processed across multiple large-scale brain networks that engage both cortical and subcortical structures (Lindquist *et al.*, 2012; Barrett and Satpute, 2013; Wager *et al.*, 2015; Pessoa, 2018). The extent to which these networks interact with brain networks specialized for other cognitive functions has also been investigated. For example, it has been proposed that emotion processing is reliant on dynamic interactions between the salience network (e.g. the amygdala and insula) and the central executive brain network for cognitive control [e.g. the lateral prefrontal cortex (PFC)] (Seeley *et al.*, 2007; Uddin, 2015; Pessoa, 2018). Both the salience network and the central executive network consist of brain areas that show greater neural activation when participants perform tasks requiring emotional processing. However, a recent model has proposed that another brain network, the default mode network (DMN) is also necessary for emotion processing (Satpute and Lindquist, 2019).

The DMN is anti-correlated with task performance, meaning it exhibits a decrease in neural activity when participants perform cognitive tasks in the fMRI scanner (Raichle, 2015). This has led to claims that the DMN mediates inner states such as mind wandering, inner thoughts and internal states (Smallwood *et al.*, 2021). fMRI studies have also demonstrated that the DMN is anti-correlated with other brain areas during facial expression naming tasks (Sreenivas *et al.*, 2012; Lanzoni *et al.*, 2020). This is consistent with the hypothesis that emotion processing is dependent on a push/pull interaction between the salience and central executive networks and the DMN (Satpute and Lindquist, 2019). Our aim in the current study was to causally test the role of the DMN in a facial expression naming task by combining fMRI with transcranial magnetic stimulation (TMS). To do this, we transiently disrupted the right inferior frontal gyrus (IFG), a brain area in the lateral PFC. Importantly, the lateral PFC contains spatially distinct brain areas that are components in different functional brain networks. The anterior parts of the lateral IFG are part of the DMN, while more posterior areas of the IFG and middle frontal gyrus (MFG) are a part of the fronto-parietal attention network (FPN) (Yeo *et al.*, 2011). The IFG has also been identified as part of the central executive network (CEN), a brain network that is anti-correlated with the DMN (Raichle, 2015).

The lateral PFC is involved in a range of different face-processing tasks including identity recognition (Ishai *et al.*, 2002), working memory for faces (Courtney *et al.*, 1996) and the configural processing of the eyes and mouth (Renzi *et al.*, 2013). Importantly, prior studies have also demonstrated that the lateral PFC is involved in facial expression processing (Gorno-Tempini *et al.*, 2001; Iidaka *et al.*, 2001). Neuropsychological studies of patients with frontal lobe damage have further demonstrated that those with lateral PFC damage have problems with a range of emotion processing tasks including theory of mind and self-emotion regulation (Tsuchida and Fellows, 2012; Jastorff *et al.*, 2016). Patient studies have also demonstrated that facial expression recognition is dependent on a wider network of visual brain areas that selectively process faces (Adolphs, 2002; Jastorff *et al.*, 2016). These include areas of the temporal cortex that are known to contain face-selective areas in both the ventral (Kanwisher *et al.*, 1997; McCarthy *et al.*, 1997) and lateral (Puce *et al.*, 1996, 1998; Gauthier *et al.*, 2000) brain surfaces. These areas have been linked together into models that propose a distributed brain network specialized for face processing (Haxby *et al.*, 2000; Calder and Young, 2005). Prior neuroimaging studies have also revealed that the lateral PFC is engaged in the top-down control of other brain areas when recognizing faces including the amygdala (Davies-Thompson and Andrews, 2012), ventral temporal cortex (Heekeren *et al.*, 2004; Baldauf and Desimone, 2014) and the superior temporal cortex (STS) (Wang *et al.*, 2020).

Our prior combined TMS/fMRI studies have causally demonstrated the connectivity between different nodes in the face-processing network. For example, we demonstrated that TMS delivered over the occipital face area (OFA) reduced the BOLD response to faces in the fusiform face area (FFA) compared to TMS delivered over a control site (Pitcher *et al.*, 2014; Groen *et al.*, 2021). TMS delivered over the face-selective area in the posterior STS reduced the BOLD response to face videos in the STS and amygdala compared to TMS delivered over the control site (Pitcher *et al.*, 2017). In addition, we compared TMS delivered over the right posterior STS and right motor cortex using resting-state fMRI (Handwerker *et al.*, 2020). Results showed that TMS delivered over STS selectively reduced functional connectivity between multiple nodes of the face network (e.g. the OFA, FFA and amygdala). Taken together, these studies demonstrate that TMS disruption of one face-selective area causes remote effects across other nodes of the face processing network. Having previously targeted face-processing areas in the occipitotemporal cortex (e.g. the OFA and STS) in the present study we disrupted the face-selective area in the right IFG (Ishai *et al.*, 2002; Nikel *et al.*, 2022).

The face-selective area in the IFG has been shown to process a range of different face-processing tasks. These include familiar face recognition (Rapcsak *et al.*, 1996), working memory for faces (Courtney *et al.*, 1997), famous-face recognition (Ishai *et al.*, 2002), processing of information from the eyes (Chan and Downing, 2011) and configural processing of the component parts of faces (e.g. the eyes and mouth) (Renzi *et al.*, 2013). Other studies have demonstrated that the IFG is involved in the top-down control of ventral temporal cortex when recognising faces (Heekeren *et al.*, 2004; Baldauf and Desimone, 2014) and is functionally connected to the amygdala (Davies-Thompson and Andrews, 2012). We therefore predicted that TMS delivered over the right PFC while participants performed a facial expression naming task would decrease neural activity across the face network. Crucially, we also predicted that transient disruption of this network would cause an increase in neural activity in the DMN because the two networks dynamically interact during facial expression naming.

## Materials and methods

### Participants

A total of 20 participants (14 females; age range 19- to 46-years old; mean age 23 years, SD = 6.4) with normal or corrected-to-normal vision gave informed consent as directed by the Ethics Committee at the University of York.

### Stimuli

Face stimuli for the expression naming task were 14 models (female and male) from Ekman and Friesen’s (1976) facial affect series expressing one of seven emotions. Each image was shown once only. This equated to a total of 110 unique pictures: anger (17), disgust (15), fear (17), happy (18), neutral (14), sad (15) and surprise (14).

In addition to the experimental task, we also ran a functional localizer to identify face-selective areas in each participant. Stimuli were 3 s movie clips of faces and objects that we have used in prior studies (Sliwinska *et al.*, 2020b, 2022; Küçük *et al.*, 2022). There were 60 movie clips for each category in which distinct exemplars appeared multiple times. Movies of faces and bodies were filmed on a black background, and framed close-up to reveal only the faces or bodies of seven children as they danced or played with toys or adults (who were out of frame). Fifteen different moving objects were selected that minimized any suggestion of animacy of the object itself or of a hidden actor pushing the object (these included mobiles, windup toys, toy planes and tractors, balls rolling down sloped inclines). Within each block, stimuli were randomly selected from within the entire set for that stimulus category. This meant that the same actor or object could appear within the same block but given the number of stimuli this did not occur regularly.

### Procedure

Participants completed three separate sessions, each performed on a different day. The first session was an fMRI experiment designed to individually localize the TMS sites in each participant. In sessions two and three, TMS was delivered over the right lateral IFG or over the vertex control site (order was balanced across participants) immediately before scanning began.

In the first session, participants viewed three runs of a functional localiser task (234 s each) to individually identify face-selective areas. Our previous fMRI study of face processing in the lateral PFC demonstrated that a face-selective area was more commonly identified across participants in the right IFG (Nikel *et al.*, 2022). Based on this study, we targeted the same location for disruption with TMS in the current study. Functional runs presented short video clips of faces, bodies and objects in 18 s blocks that contained six 3 s video clips from that category. We also collected a high-resolution structural scan for each participant.

**Fig. 1.**
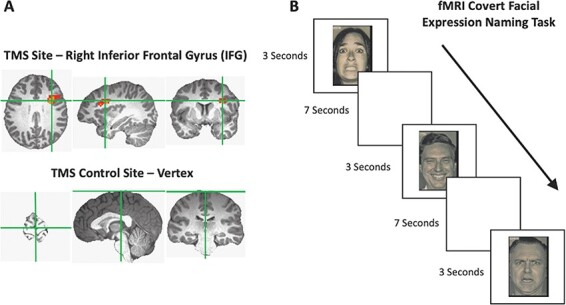
(A) The TMS sites from an example participant. The active site in the right lateral PFC was defined using a contrast of faces > objects for each participant. The average MNI coordinates (36,6,48) were centred in right IFG. (B) An example of the trial procedure for the fMRI covert facial expression naming task. Participants were required to silently name the expression on the Ekman faces that were displayed for 3 s.

Sessions two and three combined TMS and fMRI. Prior to scanning, the Brainsight TMS-MRI co-registration system (Rogue Research) was used to mark the location of the face-selective area in the right IFG based on the initial fMRI localizer data collected for each participant. The vertex control site was identified using a tape measure as a point in the middle of the head halfway between the nasion and inion (Figure 1A).

Participants were then taken to the fMRI scanner control room where thetaburst TMS was delivered over the right IFG or the vertex for each participant (stimulation site order was balanced across participants). Once stimulation was completed, participants entered the scanner room immediately. fMRI data collection began as quickly as possible as the effects of TMS are transient, but varied owing to factors like participants speed at entering the scanner. For all participants, the start of scanning began within 5 min of TMS stimulation being delivered.

Functional data for the expression naming task were acquired over two blocked-design functional runs lasting 570 s each (Figure 1B). Each run consisted of 55 trials during which facial expression stimuli were presented centrally on the screen for 3 s, followed by a blank screen of 7 s. The two runs of the expression naming task (570 s each) plus with the time taken to place the participants in the scanner (always <5 min) meant that all experimental data were collected within 30 min of TMS being delivered. Our prior studies have demonstrated that this is an effective duration to measure the impact of TMS on the BOLD signal (Pitcher *et al.*, 2014; Groen *et al.*, 2021).

Participants were instructed to silently name the emotion that the facial expression displayed when the stimuli was presented. Once the two expression naming runs were completed participants viewed two runs of the localizer task (234 s each) to individually identify face-selective areas. Functional runs presented short video clips of faces, bodies and objects in 18 s blocks that contained six 3 s video clips from that category. Once localiser data collection was completed participants exited the scanner. After the final session participants were debriefed on the nature of the study.

### Brain imaging and analysis

Imaging data were acquired using a 3 T Siemens Magnetom Prisma MRI scanner (Siemens Healthcare, Erlangen, Germany) at the University of York. Functional images were acquired with a 20-channel phased array head coil and a gradient-echo EPI sequence [38 interleaved slices, repetition time (TR) = 3 sec, echo time (TE) = 30 ms, flip angle =90 degrees; voxel size 3 mm isotropic; matrix size = 128 × 128] providing whole-brain coverage. Slices were aligned with the anterior to posterior commissure line. Structural images were acquired using the same head coil and a high-resolution T-1-weighted 3D fast spoilt gradient (SPGR) sequence [176 interleaved slices, repetition time (TR) = 7.8 s, echo time (TE) = 3 ms, flip angle = 20 degrees; voxel size 1 mm isotropic; matrix size = 256 × 256).

fMRI data were analysed using AFNI (http://afni.nimh.nih.gov/afni). Data from the first four TRs from each run were discarded. The remaining images were slice-time corrected and realigned to the last volume of the last run prior to TMS during the TMS to vertex session, and to the corresponding anatomical scan. The volume registered data were spatially smoothed with an 8 mm full-width-half-maximum Gaussian kernel. Signal intensity was normalized to the mean signal value within each run and multiplied by 100 so that the data represented percent signal change from the mean signal value before analysis.

For the localiser task a general linear model (GLM) was established by convolving the standard haemodynamic response function with two regressors of interest (faces and objects). Regressors of no interest (e.g. six head movement parameters obtained during volume registration and AFNI’s baseline estimates) were also included. Face-selective areas were identified in each participant using a contrast of faces greater than objects.

For the expression naming task, we performed two separate analyses. The first grouped all expressions together using a GLM was established by convolving the standard haemodynamic response function with one regressor of interest (faces). Regressors of no interest (e.g. six head movement parameters obtained during volume registration and AFNI’s baseline estimates) were also included in the GLM. To investigate whole-brain effects of TMS, we used a mixed effects ANOVA in AFNI (3dANOVA2) with TMS sessions (IFG and Vertex) and participants (*N* = 20) as independent factors. Group whole-brain maps were calculated for each TMS session. We then we subtracted the IFG session data from the vertex session.

In the second analysis, we performed an exploratory multi-voxel pattern analysis (MVPA) to determine whether TMS delivered over the right IFG decreases the neural discriminability between different expressions in the face network. This analysis was performed for face-selective areas identified using the functional localizer (IFG, the Amygdala, pSTS, FFA and OFA). Here, we used regions of interest (ROI) masks that included both hemispheres to increase signal-to-noise ratio in the light of the limited data available. We first created new GLMs, which contained seven regressors for each of the seven emotions (anger, disgust, fear happy, neutral, sad and surprise), separately for each fMRI run. From these GLMs, we then calculated T-maps against baseline for each emotion. The subsequent MVPA analysis was carried out using the CoSMoMVPA toolbox for Matlab (Oosterhof *et al.*, 2016). To quantify the discriminability between emotions, we used a cross-validated correlation approach (Haxby *et al.*, 2001). Specifically, we correlated (*Spearman*-correlation) response patterns (T-values across voxels in each ROI) between the two runs, either for the same emotion (within-correlations) or for different emotions (between-correlations). Subtracting the between-correlations from the within-correlations yielded a measure of neural discriminability between emotions for each ROI, separately for the two TMS sites. Discriminability in each ROI was tested against zero using one-sided *t*-tests (as below-zero values are not interpretable in this analysis). Discriminability was compared between the TMS conditions using two-sided *t*-tests.

### TMS site localization and parameters

Stimulation sites were localised using individual structural and functional images collected during an fMRI localiser task that each participant completed prior to the combined TMS/fMRI sessions. In the localiser session, participants viewed the same dynamic face and object stimuli as in earlier studies of the face network (Pitcher *et al.*, 2011; Sliwinska *et al.*, 2020a). The stimulation site targeted in the right IFG (Nikel *et al.*, 2022) of each participant was the peak voxel in the face-selective ROI identified using a contrast of greater activation by dynamic faces than dynamic objects (mean MNI co-ordinates 36,6,48). The mean MNI coordinates for all participants are included in the Supplemental Materials. The vertex site was identified as a point on the top of the head halfway between the nasion (the tip of the nose) and the inion (the point at the back of the head). TMS sites were identified using the Brainsight TMS-MRI co-registration system (Rogue Research) and the proper coil locations were then marked on each participant’s scalp using a marker pen.

A Magstim Super Rapid Stimulator (Magstim; Whitland, UK) was used to deliver the TMS via a figure-eight coil with a wing diameter of 70 mm. TMS was delivered at an intensity of 45% of machine output over each participant’s functionally localised right IFG or vertex. Thetaburst TMS (TBS) was delivered using a continuous train of 600 pulses delivered in bursts of 3 pulses (a total of 200 bursts) at a frequency of 30 Hz with a burst frequency of 6 Hz for a duration of 33.3 s and fixed intensity of 45% of the maximum stimulator output. We used a modified version (Nyffeler *et al.*, 2006) of the original thetaburst protocol (Huang *et al.*, 2005) as this version has been shown to have longer lasting effects (Goldsworthy *et al.*, 2012). The Stimulator coil handle was held pointing upwards and parallel to the midline when delivered over the right IFG and flat against the skull with handle towards the inion when delivered over the vertex.

## Results

### Whole-brain group analysis of TMS disruption of the right IFG

**Fig. 2.**
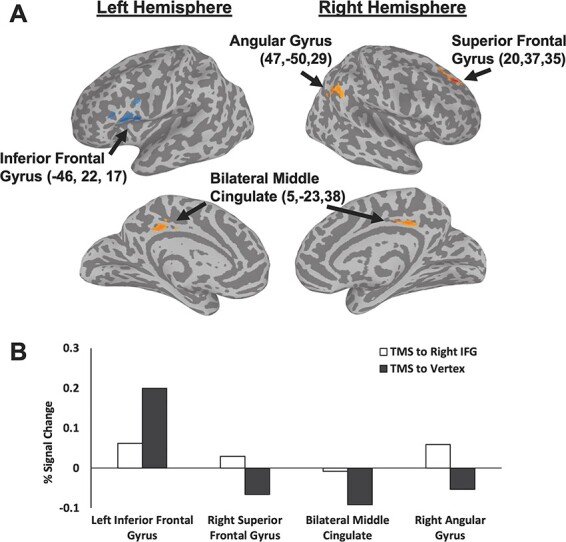
(A) The results of a group whole-brain analysis showing the distributed impact of TMS delivered over the right IFG, while participants silently named facial expressions. Group data (*N *= 20) were calculated for each TMS session and the IFG session data were then subtracted from the vertex session data (*P *= 0.005, *z*-stat = 3.1). Clusters in orange denote an increase in neural activity after TMS delivered over the right IFG. The cluster in blue denotes a decrease in neural activity after TMS delivered over the right IFG. (B) The percent signal change for the two stimulation conditions in the four regions identified in the group analysis. TMS delivered over the right IFG reduced the positive neural activity in the left IFG and increased the negative neural activity in the right SFG, right angular gyrus and bilateral middle cingulate cortex (components of the default mode network).

Experimental data (*N *= 20) from the expression naming task were entered into a group whole-brain-mixed effects analysis of variance (ANOVA) with TMS condition (right IFG and vertex control site) and participants as independent factors. Activation maps were calculated for each TMS session and the IFG session data were then subtracted from the vertex session data to establish the whole-brain effects of TMS. Data were uncorrected and thresholded (*P *= 0.005, *z*-stat = 3.1) with a cluster correction set at 50 contiguous voxels. These maps were then registered to the MNI template using probabilistic maps for combining functional imaging data with cytoarchitectonic maps (Eickhoff *et al.*, 2005). Results revealed multiple brain areas that exhibited significant differences between TMS sites (Figure 2A). TMS delivered over the right IFG compared to TMS delivered over the vertex control site reduced neural activity in the left IFG (−46, 22, 17) (133 voxels) while increasing neural activity in three nodes of the default mode network: the right superior frontal gyrus (SFG) (20, 37, 35) (90 voxels), right angular gyrus (47, −50, 29) (54 voxels) and bilateral middle cingulate cortex (5, −23, 38) (76 voxels). To determine response magnitudes against baseline, we also calculated the percent signal change for the two stimulation conditions in these four regions (Figure 2B). Consistent with the established neural response pattern of the DMN we observed a negative BOLD response in the right SFG, right angular gyrus and bilateral cingulate cortex in the vertex control condition. However, when we disrupted the right IFG the negativity of the BOLD response was reduced in all three areas compared to the vertex control condition. TMS delivered over the right IFG revealed the opposite effect on the neural activity in the left IFG. Namely, disruption of the right IFG (compared to the vertex) reduced the positive neural activity in the left IFG when performing the facial expression naming task (Figure 2B).

### ROI analysis of TMS disruption in face processing network

**Fig. 3.**
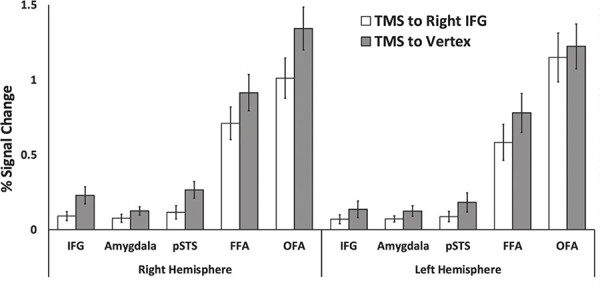
Results of the ROI analysis performed in face-selective areas for the facial expression naming task. Percent signal change (PSC) data for two stimulation conditions (right IFG and vertex) in the right and left IFG, amygdala, pSTS, FFA and OFA. Analyses revealed a significant main effect of stimulation (*P* = 0.015) in which TMS delivered over the right IFG reduced the neural response to expression naming across all nodes of the face network. There were no significant interactions. Error bars show standard errors of the mean across participants.

To further characterise the effects of disrupting the right IFG across the face-processing network, we also performed a ROI analysis at the individual participant level. ROIs were defined using the functional localiser runs from the initial fMRI session and the runs collected after the expression naming task in the combined TMS/fMRI sessions. Face-selective ROIs were identified across both hemispheres using a contrast of faces greater than objects and a statistical threshold of *P* < 0.1. This was based on our prior study of the face-selective areas in the bilateral IFG which demonstrated that this threshold was necessary (Nikel *et al.*, 2022). We identified clusters of at last 5 voxels in each defined face area and created a 5 mm sphere around the peak activation coordinate for the following ROIs in both hemispheres: IFG, Amygdala, posterior superior temporal sulcus (pSTS), fusiform face area (FFA) and occipital face area (OFA). We then calculated the percent signal change for the two stimulation conditions in each ROI (Figure 3).

The percent signal change (PSC) data for the expression naming task for the two stimulation conditions were entered into a 2 (stimulation: Right IFG, Vertex) by 2 (Hemisphere: right, left) by 5 (ROI: IFG, amygdala, pSTS, FFA, OFA) repeated measures ANOVA. Results showed significant main effects of stimulation [*F* (1,19) = 7.2, *P* = 0.015; partial η^2^ =0.275] and ROI [*F* (4,76) = 66.9, *P* < 0.001; partial η2 =0.779] but not of hemisphere [*F* (1,19) = 1.2, *P* = 0.27; partial η^2^ =0.063]. There was no significant three-way interaction between stimulation, hemisphere, and ROI [*F* (4,76) = 1.9, *P* = 0.112; partial η^2^ =0.09]. All three of the two-way interactions were also non-significant (*P > *0.065).

### Multivoxel pattern-wide analysis analysis

Finally, we performed an exploratory multivoxel pattern-wide analysis (MVPA) on the facial expression data. It is worth noting that the amount of data used for MVPA here is less than the amount of data used in fMRI only studies because we only collected data during the duration when we expected TMS to disrupt activity. The MVPA can establish whether TMS delivered over the right IFG selectively impaired the neural discriminability of the facial expressions presented (anger, disgust fear, happy, neutral, sad and surprise). Emotions could be discriminated from activity patterns in the bilateral pSTS, both after TMS over IFG [*t* (19) = 3.14, *P* = 0.003] and vertex [trending at *t*(19) = 1.59, *P* = 0.06]. Emotions were also discriminable from activity patterns in the bilateral FFA [trending at *t*(19) = 1.37, *P* = 0.09] and OFA [*t*(19) = 2.86, *P* = 0.005], but only after TMS over vertex. The OFA was the only region showing a TMS-related difference: emotions were more readily discriminable from OFA response patterns when TMS was applied over vertex compared to TMS over the IFG [*t*(19) = 2.17, *P* = 0.04]. We further investigated whether the effect in OFA was specifically driven by changes in the representation of negative or positive/neural emotions. However, repeating the MVPA for the negative (anger, disgust, fear, sadness) or positive/neural (neutral, happiness, surprise) emotions separately, we did not find any differences in emotion discrimination between TMS over vertex and TMS over the IFG [negative: *t*(19) = 1.11, *P* = 0.28; positive/neural: *t*(19) = 0.95, *P *= 0.35]. While this could suggest that there is no modulation of discrimination within emotion categories, our limited scan time may not offer enough statical power to separately assess positive and negative emotions.

## Discussion

In the current study, participants were scanned using fMRI during two separate sessions while performing a facial expression naming task. Prior to scanning TMS was delivered over a functionally localised face-selective area centered at the right IFG, or over the vertex control site. We then calculated the changes in neural activity by subtracting the the BOLD data collected during the right IFG stimulation session from the BOLD data collected during the vertex stimulation condition. The results of a whole-brain group analysis (Figure 2) demonstrated that TMS delivered over the right IFG decreased neural activity in the left inferior frontal gyrus (compared to when TMS was delivered over the vertex). The same analysis also revealed an increase in neural activity in three nodes of the DMN: the right SFG, right angular gyrus and the bilateral middle cingulate gyrus. The ROI analysis of the face-selective areas revealed a main effect of stimulation. TMS delivered over the right IFG reduced the neural response across all bilateral face ROIs compared to the vertex control condition (Figure 3). Our results are consistent demonstrate that visually naming facial expressions involves an interaction a dynamic push/pull interaction between the face-processing network (Haxby *et al.*, 2000; Calder and Young, 2005) and the DMN (Raichle, 2015; Smallwood *et al.*, 2021). This is consistent with a recent a combined TMS/EEG study that demonstrated a dynamic interaction between the inferior frontal cortex and the DMN during action performance task (Zanon *et al.*, 2018).

Our prior studies that combined TMS and fMRI also demonstrated distributed disruption across the face network (Pitcher *et al.*, 2014, 2017; Handwerker *et al.*, 2020; Groen *et al.*, 2021). The lack of an interaction between stimulation site and ROI (Figure 3) suggests that all five ROIs in both hemispheres are connected to the IFG during facial expression naming. This is consistent with patient and TMS studies showing that the IFG (Paracampo *et al.*, 2017, 2018; Penton *et al.*, 2017), pSTS (Pitcher, 2014; Sliwinska *et al.*, 2020b), FFA (Rezlescu *et al.*, 2012), OFA (Pitcher *et al.*, 2008) and the amygdala (Adolphs *et al.*, 1994) are all involved in facial expression recognition. Our findings reveal that the right IFG is directly or indirectly connected to all other regions in the face network. This is consistent with prior studies demonstrating that lateral prefrontal cortex is implicated in a range of neural processes that including cognitive control (MacDonald *et al.*, 2000), working memory (Curtis and D’Esposito, 2003), and Theory of Mind (Kalbe *et al.*, 2010), executive function (Goldman-Rakic, 1996, 2000) and the top-down of visual recognition (Heekeren *et al.*, 2004; Baldauf and Desimone, 2014).

The results of the group whole-brain analysis revealed that TMS delivered over the right IFG (compared the vertex control site) reduced neural activity in the left IFG for the expression naming task. The right IFG was selected as the TMS stimulation site because our prior study had demonstrated a greater response to visually presented faces in the right, more than the left IFG (Nikel *et al.*, 2022). Despite this lateralisation, the left frontal cortex has still been implicated in a range of face processing tasks. For example, prior fMRI studies have demonstrated that the left IFG exhibits greater activity in facial expression recognition tasks (Gorno-Tempini *et al.*, 2001; Trautmann *et al.*, 2009; Regenbogen *et al.*, 2012). In addition, other tasks such as evaluating the social impact of facial expressions (Prochnow *et al.*, 2014) and facial expression matching tasks (Sreenivas *et al.*, 2012) also generate greater activity in the left IFG. The reduction in neural activity in the current study may have also been partially driven by the silent naming task participants performed. This would be consistent with the established role of the left IFG as a ‘high-level’ language brain area (Fedorenko and Thompson-Schill, 2014; Fedorenko and Blank, 2020). More generally, our data show that face networks in both hemispheres are tightly interconnected, where disruption of one network node (the right IFG) has consequences on the activity in contralateral nodes like the left IFG.

We also performed an exploratory MVPA analysis to establish whether TMS disruption of the right IFG disrupted the neural representation of emotions. This analysis revealed that TMS over the IFG reduced the neural discriminability of emotions in OFA, but not any of the other regions. This suggests that TMS to the IFG can disrupt emotion processing in areas of the core face network. It is worth noting that this result was obtained under experimental conditions that are suboptimal for MVPA: the temporally constrained nature of TBS effects (lasting for only about 30 min; (Pitcher *et al.*, 2014, 2017; Handwerker *et al.*, 2020; Groen *et al.*, 2021) drastically reduces the amount of available fMRI data, compared to typical MVPA studies of emotion processing (Said *et al.*, 2010; Harry *et al.*, 2013; Wegrzyn *et al.*, 2015). Whether the effect observed in the OFA here truly extends to a larger set of areas, perhaps including the FFA, could be tested in future studies that use concurrent fMRI/TMS approaches (Mizutani-Tiebel *et al.*, 2022) to increase the amount of available data. A surprising result in our MVPA is that pSTS, which is often considered a key region for emotion discrimination, did not show altered emotion representations after TMS to the IFG. Future studies should investigate whether such effects appear when emotion processing is probed with dynamic stimuli, which are strongly preferred by the region (Pitcher and Ungerleider, 2021). It is worth highlighting that the results of our MVPA should be interpreted with caution. As they were obtained with very little available data for multivariate analyses, and as the effects are statistically not very robust, they only provide a first benchmark of how facial emotion processing could change after PFC disruption. Further studies are needed to solidify our results. For example, it is unclear whether studies with adequate power will be able to distinguish between different emotional expressions when accounting for factors such as valence.

The overall pattern of our results demonstrates that naming facial expressions is dependent on the interaction of different functional brain networks. While it is common for researchers to talk about the face-processing network (Haxby *et al.*, 2000) it is also important to note that the nodes of this network are distributed across brain areas with different cognitive functions. These include visual areas in occipito-temporal cortex (FFA, OFA, pSTS), emotion processing areas (the amygdala) and cognitive control areas (IFG). The results of the current study demonstrate the push/pull dynamic network interactions between these brain areas and nodes in the DMN. This is consistent with models of that proposing that emotion processing is a complex process that is dependent on the interactions of brain networks with different cognitive functions (Uddin, 2015; Pessoa, 2018; Satpute and Lindquist, 2019).

## Supplementary Material

nsad072_Supp

## Data Availability

The data underlying this article will be shared on reasonable request to the corresponding author.
